# Supplementary material for: Growing up is hard to do: a demographic model of survival and growth of Caribbean octocoral recruits
Source: PeerJ. 2022 Nov 18;10:e14386. doi: 10.7717/peerj.14386 (PMC9677878; doi:10.7717/peerj.14386)
Supplement: Supplemental Information 1 [file peerj-10-14386-s001.docx]

Supplemental Material

Supplemental Table 1. Sensitivity and elasticity of the empirical model of recruit growth and survival.

**Proportion of colonies attaining 5 cm height**

Sensitivity

Size Class 0.3 - 0.5 0.6 - 1.1 1.2 - 2.0 2.1 - 5.0

0.3 - 0.5 2.100 0.603 0.202 0.294

.6 - 1.1 3.284 0.977 0.327 0.483

1.2 - 2 5.551 1.652 0.567 0.842

2.1 - 5.0 18.503 5.618 1.927 3.014

>5 128.051 39.363 13.608 21.431

Elasticity

Size Class 0.3 - 0.5 0.6 - 1.1 1.2 - 2.0 2.1 - 5.0

0.3 - 0.5 0.252 0.020 0.001 0.003

.6 - 1.1 0.414 0.142 0.025 0.023

1.2 - 2 0.167 0.109 0.055 0.020

2.1 - 5.0 0.445 0.333 0.269 0.809

>5 * * * *

**Time (years) to reach 5 cm**

Sensitivity

Size Class 0.3 - 0.5 0.6 - 1.1 1.2 - 2.0 2.1 - 5.0

0.3 - 0.5 0.493 0.591 0.254 0.562

.6 - 1.1 0.318 0.696 0.314 0.750

1.2 - 2 0.039 0.785 0.412 1.069

2.1 - 5.0 -0.535 0.475 0.371 1.646

>5 -0.825 -1.664 -0.803 0.000

Elasticity

Size Class 0.3 - 0.5 0.6 - 1.1 1.2 - 2.0 2.1 - 5.0

0.3 - 0.5 0.036 0.012 0.001 0.004

.6 - 1.1 0.024 0.062 0.015 0.021

1.2 - 2 0.001 0.032 0.025 0.015

2.1 - 5.0 -0.008 0.017 0.032 0.269

>5 * * * *

* Elasticity, which is a measure of proportionate change cannot be calculated when the original transition matrix value is zero
